# Chloroquine and Hydroxychloroquine Interact Differently with ACE2 Domains Reported to Bind with the Coronavirus Spike Protein: Mediation by ACE2 Polymorphism

**DOI:** 10.3390/molecules26030673

**Published:** 2021-01-28

**Authors:** Riadh Badraoui, Mohd Adnan, Fevzi Bardakci, Mousa M. Alreshidi

**Affiliations:** 1 Laboratory of General Biology, Department of Biology, University of Ha’il, Ha’il 81451, Saudi Arabia; mo.adnan@uoh.edu.sa (M.A.); fbardakci@adu.edu.tr (F.B.); mousa.algladi@gmail.com (M.M.A.); 2 Section of Histology—Cytology, Medicine College of Tunis, University of Tunis El Manar, Djebel Lakhdhar Road, La Rabta-Tunis 1007, Tunisia; 3 Laboratory of Histo-Embryology and Cytogenetic, Medicine College of Sfax, University of Sfax, Majida Boulila Road, Sfax 3029, Tunisia; 4 Laboratory of Genetics, Department of Biology, Aydin Adnan Menderes University, Aydin 09010, Turkey; 5 Molecular Diagnostic and Personalized Therapeutics Unit, University of Ha’il, Ha’il 81451, Saudi Arabia

**Keywords:** ACE2 allelic variants, chloroquine, hydroxychloroquine, molecular interactions, coronavirus, binding domain, molecular docking, in silico

## Abstract

The severe acute respiratory syndrome coronavirus 2 (SARS-CoV-2) infection inducing coronavirus disease 2019 (COVID-19) is still an ongoing challenge. To date, more than 95.4 million have been infected and more than two million deaths have been officially reported by the WHO. Angiotensin-converting enzyme (ACE) plays a key role in the disease pathogenesis. In this computational study, seventeen coding variants were found to be important for ACE2 binding with the coronavirus spike protein. The frequencies of these allele variants range from 3.88 × 10^−3^ to 5.47 × 10^−6^ for rs4646116 (K26R) and rs1238146879 (P426A), respectively. Chloroquine (CQ) and its metabolite hydroxychloroquine (HCQ) are mainly used to prevent and treat malaria and rheumatic diseases. They are also used in several countries to treat SARS-CoV-2 infection inducing COVID-19. Both CQ and HCQ were found to interact differently with the various ACE2 domains reported to bind with coronavirus spike protein. A molecular docking approach revealed that intermolecular interactions of both CQ and HCQ exhibited mediation by ACE2 polymorphism. Further explorations of the relationship and the interactions between ACE2 polymorphism and CQ/HCQ would certainly help to better understand the COVID-19 management strategies, particularly their use in the absence of specific vaccines or drugs.

## 1. Introduction

**Figure 1:**
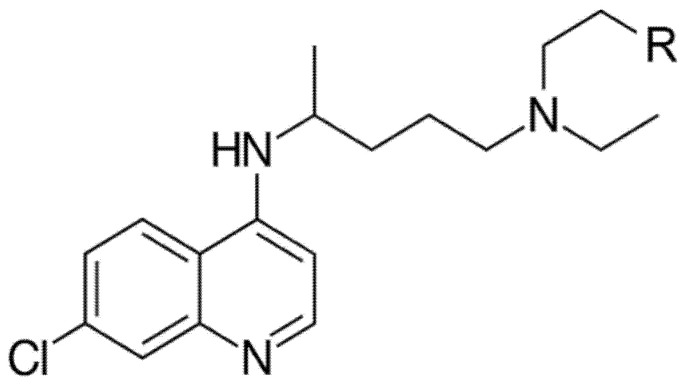
Chemical structure of chloroquine (CQ, R=H) and hydroxychloroquine (HCQ, R=OH).

Chloroquine (CQ) and its metabolite hydroxychloroquine (HCQ) (Figure 1) are mainly used to prevent and treat malaria and rheumatic diseases (including rheumatoid and idiopathic arthritis and systemic lupus erythematous), respectively [1]. Recently, Xu et al. (2018) [2] reported efficient effects of CQ and HCQ in the treatment of cancer via autophagy inhibition. The half-life of HCQ is about one month and it takes about six months for a full elimination from the body [3]. CQ and HCQ act as chemotherapeutic agents against erythrocytic case plasmodium parasites following malarial infection. Both increase the pH of the parasite’s vacuole leading to disruption of its development and asexual reproduction [4].

The severe acute respiratory syndrome coronavirus 2 (SARS-CoV-2) started in Wuhan China. It has caused the worldwide COVID-19 (coronavirus disease 2019) pandemic. Currently, there are no specific drugs or vaccines available and people are still dying mainly with acute respiratory distress syndrome (ARDS) which is one of the main severe complications of COVID-19 [5]. Throughout the ongoing COVID-19 pandemic, the use of CQ and HCQ has been allowed in several countries to treat the SARS-CoV-2 infected people. It has been reported that both CQ and HCQ interfere with various cellular levels and might have a wide range of antiviral potencies even on cancer cells [2,6,7]. In fact, both inhibit the SARS-CoV-2 viral replication [8], decrease antigen processing and its presentation [9,10], and decrease the cellular activity via low secretion of inflammatory cytokines and type 1 interferon [5]. CQ and HCQ might also interfere with angiotensin-converting enzyme 2 (ACE2) receptor which is involved in COVID-19 and its symptoms [11]. Strong interactions have been reported between the SARS-CoV-2 RBD domain of the S protein and ACE2 [12]. In fact, SARS-CoV-2 binds, and then invades the target cells through ACE2 [13]. So far, cells highly expressing ACE2 such as lung, kidney, and vascular endothelial cells may be targeted by SARS-CoV-2 [14,15].

While ACE1 and ACE2 showed only 42% amino acid similarity, both cleave amino acids from the C-terminal chain of peptides [16]. It has been reported that ACE polymorphism might play a crucial role in the pathogenesis of COVID-19 using a multiple regression model [17,18]. Several studies have reported that some drugs, including CQ, HCQ, remdesivir, and favipiravir are useful in COVID-19 treatment strategies but, currently, two vaccine types, including an mRNA-based new technology, are authorized and recommended for use and several vaccines are undergoing large-scale (phase 3) clinical trials [19,20,21]. There has been no evidence of the benefits of using CQ and HCQ in the therapy of COVID-19. However, it is worthwhile to assess the interaction modes with various targets of interest, which may be used for the future design of new drugs or new diseases. In fact, recently there was a clear interest in these drugs during the ongoing COVID-19. A recent study reported the interaction of CQ and HCQ with ACE2. Similarly, some phytochemical compounds displayed potential therapeutic targets of COVID-19 via inhibition of ACE2 [22,23]. As far as we know, to date, no study has reported the different interactions of these drugs, particularly both CQ and HCQ, with the various ACE2 domains reported to bind with coronavirus spike protein and the mediation by ACE2 polymorphism. Hence, the present study aimed at identifying the different ACE2 variants which may interact with the SARS-CoV-2 virus and investigate their potential interactions with both CQ and HCQ using computational approaches. An overview is also given about the pharmacokinetics of CQ and HCQ using an ADME (absorption, distribution, metabolism and excretion) approach.

## 2. Materials and Methods

### 2.1. Structure and Genetic ACE2 Polymorphism

The genetic variants of human ACE2 and the allele frequencies were collected from Ensembl Genome Browser [24,25] and gnomAD [26]. Appropriate filters were used to select only the coding region of the different ACE2 variants. Accordingly, the coding region of seventeen variants of ACE2 gene that have been previously reported to bind with both SARS-CoV and SARS-CoV-2 [27,28] were selected for this study (Table 1). The corresponding protein sequences of human ACE2 (Q9BYF1) were retrieved from UniProt. Structures of the proteins were identified by PDB-BLAST and obtained from the RCSB protein data bank [29]. The amino acid changes and the allele frequencies were assessed.

### 2.2. In Silico Approach of ADME, Pharmacokinetics, and Docking Study

Each of the seventeen ACE2 variant receptors was used separately to assess its interactions with both chloroquine (CQ) and hydroxychloroquine (HCQ). The three-dimensional (3D) structures of CQ and HCQ were retrieved from the PubChem website (CID 2719 and CID 3652, respectively). SMILES notations were used for assessment of the pharmacokinetic and ADME parameters. The physicochemical and pharmacokinetics properties were assessed and compared. ADME (absorption, distribution, metabolism and excretion) characteristics were checked using SwissADME. AutoDock Vina was used for the generation of the different binding poses based on the CHARMM force field [30]. The different variants were prepared; water molecules and heteroatoms were removed. Then, the processed proteins with polar hydrogens and Coleman charges were used to generate different poses. Regarding chirality, (*S*)-enantiomers particularly *S*-13a, of both CQ and HCQ were used. Redocking was performed to check the efficiency of the docking assay. The predicted binding affinity and the intermolecular bonds were monitored and analyzed. The intermolecular bonds including conventional hydrogen bonds, carbon-hydrogen bonds, alkyl, Pi-alkyl, halogen, and van der Waals were explored using DS visualizer 2016.

## 3. Results and Discussion

The global COVID-19 pandemic is still an ongoing challenge because SARS-CoV-2 infection constitutes a serious threat both to human life and socioeconomic development [5]. Two vaccine types are authorized and recommended for use and some other COVID-19 vaccines are undergoing large-scale (phase 3) clinical trials [20]. In this study, the genetic variants of human ACE2 and the allele frequencies were collected from Ensembl Genome Browser [24,31] and gnomAD [26]. Seventeen coding variants of ACE2 were found to bind with the coronavirus spike protein. The interactions of CQ and HCQ with these ACE2 domain variants is well mediated by ACE2 polymorphism. Recognition of these interactions might be useful for better prognostic or shortening the recovery time in COVID-19 hospitalized patients. In fact, some COVID-19 useful drugs have been reported to shorten the time of recovery in United States hospitalized patients infected with SARS-CoV-2 [21,32].

### 3.1. ACE2 Coding Variants

Human ACE2 protein contains 805 amino acids and has two functional domains, i.e., N-terminal peptidase M2 domain and C-terminal collectrin domain, which have been reported to contain the residues involved in the spike protein binding [27,33]. This binding site is considered to be an entry door for the virus and several vaccine approaches are based on shutting this entry door in the host cells to combat this unprecedented pandemic [34]. Ensembl Genome Browser and gnomAD exhibited 345 and 242 natural ACE2 coding variants, respectively. Nevertheless, only seventeen coding variants were found to be important for ACE2 binding with the coronavirus spike protein (Table 1). The frequencies of these allele variants range from 3.88 × 10^−3^ to 5.47 × 10^−6^ for rs4646116 (K26R) and rs1238146879 (P426A), respectively. These results parallel recent published findings [28,35], in which the authors reported some rare and common ACE2 variants susceptible to SARS-CoV-2 infection. The variant rs4646116 (K26R) has been reported to be the most frequent in the Ashkenzai Jewish population [36]. These frequencies may explain the infection rate for this highly contagious virus but also the possible non-strong relationship between ACE2 variants and COVID-19 severity in different populations [36,37].

### 3.2. Molecular Binding and Interaction Results

**Figure 2:**
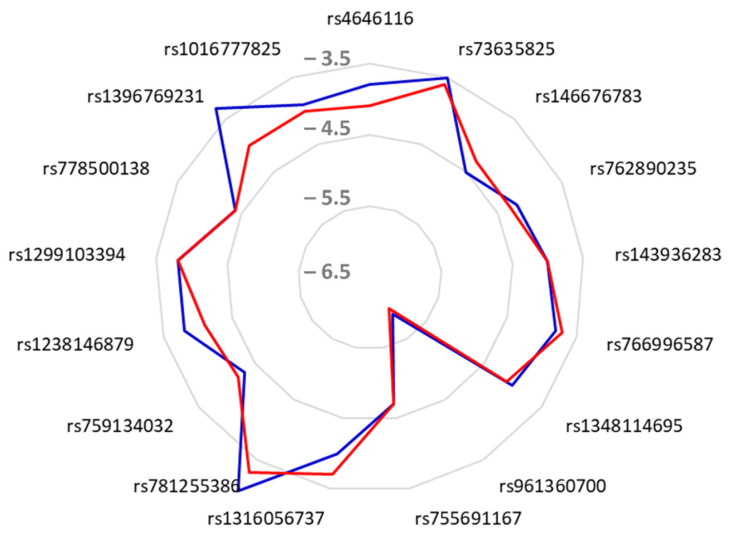
Radar distribution of chloroquine (CQ, blue color) and hydroxychloroquine (HQC, red color) binding energy towards the different variants of human ACE2. Note the superposition only in 4 allelic variants, i.e., rs143936283 (E329G), rs755691167 (K68E), rs1299103394 (K26E), and rs778500138 (E35D).

In this study, a comparison of the different binding scores of CQ and HCQ with the different allelic variant of ACE2 is reported. Table 2 shows the predicted binding affinities of the stable ACE2 variant–CQ or –HCQ complexes, number of conventional H-bonds, and the number of the closest interacting residues. Both CQ and HCQ were found to exhibit negative binding energy, ranging from −6 to −3 kcal·mol^−1^, with the different ACE2 allelic variants. Accordingly, all complexes of ACE2 variants and CQ or HCQ displayed negative docking scores. Thus, the disruption of coronavirus entry via ACE2 is thermodynamically possible by using CQ or HCQ. Further analyses using molecular dynamic approaches would confirm our results. Both CQ and HCQ interact differently with the seventeen different targeted ACE2 domains, which had been reported to bind with coronavirus spike protein. It could be deduced that CQ and HCQ efficiency might be mediated by the ACE2 polymorphism, as their interactions depend on the latter. In this study, (*S*)-enantiomers particularly *S*-13a of both CQ and HCQ were used for the molecular docking assay. In fact, it has been previously reported that (*S*)-enantiomers are consistently showing better activity than corresponding (*R*)-enantiomers, especially the antimalarial effects of CQ and its analogues [38]. The best affinity was predicted for the variant 8 (rs961360700, D355N) by −6 and −5.9 kcal·mol^−1^ for HCQ and CQ, respectively. The radar distribution of CQ and HCQ binding affinities towards the allelic variants of ACE2 showed superposition only in four alleles which are rs762890235 (P389H), rs755691167 (K68E), rs1299103394 (K26E), and rs778500138 (E35D) (Figure 2). Recently, it has been reported that CQ and HCQ also interact differently with fifteen protein targets of SARS-CoV-2 using molecular docking and dynamics [39]. This can interfere with the inhibitory activity of ACE2, which has been previously reported [22]. In this study, we highlight ACE2 polymorphism as possible interference with CQ and HCQ.

However, none of these superposed points was associated with a similar number of conventional hydrogen bonds or ACE2 interacting residues. It could be deduced that the terminal hydroxyl group, which makes the difference between CQ and HCQ, is conditioning and playing a marked influence in the binding affinities, number of conventional hydrogen bonds, and the interacting residues. This could confirm previous data of Fantini et al. (2020) [40] who reported differential interactions of CQ and HCQ with sialic acids, which is also used by the S protein of SARS-CoV-2 as an entry receptor. Recently, it was reported that some ACE2 variants decreased and some others increased the electrostatic attraction towards SARS-CoV-2, such as ACE2-K26R and ACE2-R219C [36]. Likewise, this study outlined that ACE2 variants interact differently with CQ and HCQ.

Nevertheless, the number of conventional H-bonds and the number of the closest interacting residues were slightly better with HCQ. In fact, regarding conventional hydrogen bonds (H-bonds), which constitute the best bond and the most used category in drug design [41,42], the highest number (n = 5) was found in the ACE2 (variant 16: rs1396769231, M383T)–HCQ complex. That was followed by ACE2 (variant 12: rs1299103394, K26E)–CQ, ACE2 (variant 6: rs766996587, M82I)–CQ, and ACE2 (variant 8: rs961360700, D355N)–HCQ complexes with four H-bonds each (Table 3).

ACE facilitate the invasion of the virus and its depletion from the cell membrane enhance the damaging effects, which result in tissue deterioration, particularly, in the respiratory tract. Similarly, the genetic variation within ACE2 polymorphism might result in various effects of the virus on the targeted tissues. Likewise, CQ and HCQ might interact differently with ACE2 variants.

This could be correlated with the geographical distribution of ACE2 genotype which has been previously reported [43]. For its entry within the cell, SARS-CoV-2 uses both ACE2 and the ganglioside-attached sialic acids [5,40]. Further studies on the interactions of CQ and HCQ with ganglioside-attached sialic acids could give general ideas about the possible actions of these drugs on the virus entry.

### 3.3. Pharmacokinetics and ADME Findings of CQ and HCQ

Pharmacokinetics and the most pertinent absorption, distribution, metabolism, and excretion (ADME) parameters of both CQ and HCQ were also assessed based on their absorption, distribution, metabolism, and excretion data. Table 4 exhibits the properties, lipophylicity, druglikeness, and pharmacokinetics of CQ and HCQ. 

Both drugs obey the Lipinski’s rule and show comparable and almost similar properties associated with good oral absorption and bioavailability score (0.55), low TPSA values, and acceptable consensus Log Po/w, which allow them to fall in the pink colored zone of the polygon suggesting good oral bioavailability (bottom of Table 4). Regarding pharmacokinetics results, both CQ and HCQ were predicted to have high gastrointestinal (GI) absorption and were blood–brain–barrier (BBB) permeant. Neither of the two drugs is a substrate of the p-glycoprotein. Cytochrome P450 (CYP) isoforms, such as CYP1A2, CYP2C19, CYP2C9, CYP2D6, and CYP3A4 are commonly used in biotransformation of drugs and xenobiotics [44]. In this study, the screened CYP enzyme isoforms data indicate that both CQ and HCQ were found to be inhibitors of only three isoenzymes, i.e., CYP1A2, CYP3A4, and CYP2D6 isoforms. Both CQ and HCQ displayed negative skin permeability, thus, both are not suitable compounds for transdermal delivery.

Taken together, the pharmacokinetic and ADME properties of CQ and HCQ complications such as heart failure and several non-reversible disorders have been previously, reported [45]. Our ADME and pharmacokinetic results confirmed previous data about pharmacokinetics and pharmacogenomics of CQ and HCQ [46]. The importance of the pharmacokinetic, ADME, and QSAR (for quantitative structure–activity relationship) assays was previously largely reported in the design and the assessment of several drugs [41,47,48]. 

The reported CQ and HCQ interactions with the different ACE2 variants known to bind with SARS-CoV-2 might certainly explain the variety of responses and new mechanistic effects of these drugs. In fact, new mechanisms of actions still need to be further explored [41]. The allelic variation of ACE2 may affect the recognition and infection by SARS-CoV-2, and therefore the susceptibility to its causing disease (COVID-19), and also to the treatment [49]. The current study proved that ACE polymorphism might mediate both the infection and the treatment of COVID-19. The promising effects of both CQ and HCQ that have been reported by treating the virus infection via blocking the binding of the virus with ACE2 confirms our interactions’ results [5,11]. Nevertheless, this effect might be mediated by ACE2 polymorphism. While CQ and HCQ clinically showed no potential beneficial effect on COVID-19, their interaction with ACE2 variants may certainly be used for the future design of new drugs or new diseases.

## 4. Conclusions

In conclusion, both CQ and HCQ interact differently with the different targeted ACE2 domains, which have been reported to bind with the coronavirus spike protein. It could be deduced that CQ and HCQ efficiency might be mediated by the ACE2 polymorphism. By extrapolation, the selection of CQ or HCQ for SARS-CoV-2 infected patients may be based on the ACE2 allelic variants to guarantee a more efficient drug. Further explorations of the relationship and the interactions between ACE2 polymorphism and CQ/HCQ would certainly help to better understand the COVID-19 management strategies and shorten the recovery period, particularly, in the absence of specific vaccines or drugs. This would certainly contribute to avoiding CQ and HCQ complications such as acute toxicity, heart failure, and several non-reversible disorders which have been previously reported [45,50]. The results of the current study provide new and strong evidence regarding COVID-19 susceptibility and treatment as a result of the ACE2 polymorphism.

## Figures and Tables

**Table 1:** Genetic variants change amino acids and allele frequencies of human angiotensin-converting enzyme 2 (ACE2) reported to bind with coronavirus.

Variant No.	Genetic Variant	Amino Acid Change	Allele Frequency
1	rs4646116	K26R	3.88 × 10^−3^
2	rs73635825	S19P	3.13 × 10^−4^
3	rs146676783	E37K	3.9 × 10^−5^
4	rs762890235	P389H	3.83 × 10^−5^
5	rs143936283	E329G	3.44 × 10^−5^
6	rs766996587	M82I	2.44 × 10^−5^
7	rs1348114695	E35K	1.64 × 10^−5^
8	rs961360700	D355N	1.17 × 10^−5^
9	rs755691167	K68E	1.09 × 10^−5^
10	rs1316056737	D427Y	1.09 × 10^−5^
11	rs781255386	T27A	1.09 × 10^−5^
12	rs1299103394	K26E	5.45 × 10^−6^
13	rs759134032	P84T	5.47 × 10^−6^
14	rs1238146879	P426A	5.47 × 10^−6^
15	rs778500138	E35D	N/A
16	rs1396769231	M383T	N/A
17	rs1016777825	R559S	N/A

**Table 2:** Ligand receptor interactions between chloroquine or hydroxychloroquine and the different variants of human ACE2.

No.	Genetic Variant	Chloroquine (CQ)			Hydroxychloroquine (HCQ)		
		Affinity (Kcal/Mol)	Conventional H-Bonds	Number of Closest Interacting Residues	Affinity (Kcal/mol)	Conventional H-Bonds	Number of Closest Interacting Residues
1	rs4646116	−3.8	2	4	−4.1	2	4
2	rs73635825	−3.5	3	3	−3.6	3	3
3	rs146676783	−4.5	2	4	−4.3	2	4
4	rs762890235	−4.2	1	7	−4.3	3	3
5	rs143936283	−4.0	1	6	−4.0	2	5
6	rs766996587	−3.8	4	3	−3.7	3	3
7	rs1348114695	−4.0	1	5	−4.1	2	5
8	rs961360700	−5.9	2	6	−6.0	4	7
9	rs755691167	−4.7	2	4	−4.7	2	6
10	rs1316056737	−4.0	2	3	−3.7	3	7
11	rs781255386	−3.0	1	4	−3.3	3	3
12	rs1299103394	−3.8	4	2	−3.8	4	4
13	rs759134032	−4.3	2	6	−4.2	2	5
14	rs1238146879	−3.8	2	5	−4.1	3	6
15	rs778500138	−4.4	2	4	−4.4	3	2
16	rs1396769231	−3.3	3	6	−4.0	5	6
17	rs1016777825	−3.9	2	3	−4.0	2	4

**Table 3:** The best interacting complexes of ACE2 variants’ active site residues and CQ or HCQ.

	Receptor-Ligand Interactions, Distance in Angstroms and 2D Interactions Diagrams	Receptor-Ligand 3D interaction Microphotographs
Chloroquine (CQ)	**rs1299103394 (K26E)—CQ** (CYS16)---(CQ) Conventional hydrogen bond: 3.702 Å; (ILE27)---(CQ) Conventional hydrogen bond: 2.269 Å; (SER10)---(CQ) Conventional hydrogen bond: 2.849 Å; (PHE15)---(CQ) Conventional hydrogen bond: 3.572 Å; (ALA18)---(CQ) Alkyl interaction: 4.047 Å; (CYS16)---(CQ) Alkyl interaction: 4.802 Å; (TRP37)---(CQ) Pi-Alkyl interaction: 5.195 Å. 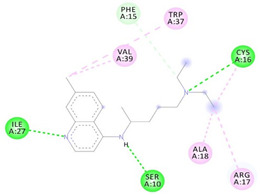	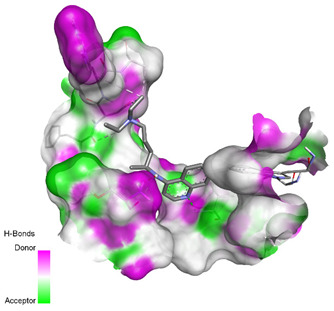
	**rs766996587 (M82I)—CQ** (TYR33)---(CQ) Conventional hydrogen bond: 2.227 Å; (GLN51)---(CQ) Conventional hydrogen bond: 1.983 Å; (TYR33)---(CQ) Conventional hydrogen bond: 3.385 Å; (TYR33)---(CQ) Conventional hydrogen bond: 3.361 Å; (PRO34)---(CQ) Pi-Sigma interaction: 3.437 Å; (TYR33,PRO34)---(CQ) Amide-Pi Stacked interaction: 4.757 Å; (PRO34)---(CQ) Pi-Alkyl interaction: 5.295 Å. 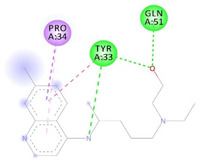	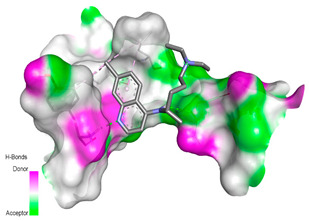
Hydroxychloroquine (HCQ)	**rs1396769231 (M383T)—HCQ** (GLY28)---(HCQ) Conventional hydrogen bond: 2.900 Å; (SER48)---(HCQ) Conventional hydrogen bond: 2.271 Å; (SER48)---(HCQ) Conventional hydrogen bond: 3.177 Å; (GLY28)---(HCQ) Conventional hydrogen bond: 3.296 Å; (SER46)---(HCQ) Conventional hydrogen bond: 3.399 Å; (PRO53)---(HCQ) Alkyl interaction: 5.246 Å; (LYS27)---(HCQ) Alkyl interaction: 4.494 Å; (ARG49)---(HCQ) Pi-Alkyl interaction: 5.201 Å; (PRO53)---(HCQ) Pi-Alkyl interaction: 5.246 Å; (PRO53)---(HCQ) Pi-Alkyl interaction: 3.918 Å. 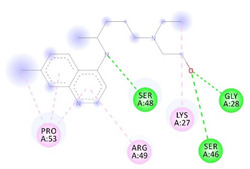	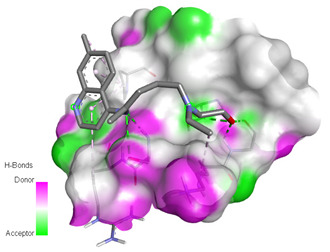
	**rs961360700 (D355N)—HCQ** (THR26)---(HCQ) Conventional hydrogen bond: 2.344 Å; (THR26)---(HCQ) Conventional hydrogen bond: 2.537 Å; (HIS24)---(HCQ) Conventional hydrogen bond: 3.377 Å; (THR26)---(HCQ) Conventional hydrogen bond: 3.270 Å; (MET11)---(HCQ) Carbon hydrogen bond: 3.494 Å; (VAL22)---(HCQ) Carbon hydrogen bond: 3.455 Å; (TRP28)---(HCQ) Pi-Hydrogen bond: 3.018 Å; (ALA21)---(HCQ) Alkyl interaction: 3.885 Å; (MET11)---(HCQ) Alkyl interaction: 4.566 Å; (LEU30)---(HCQ) Pi-Alkyl interaction: 5.174 Å. 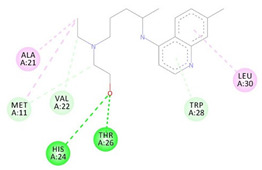	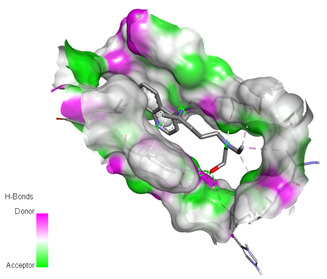

**Table 4:** Physicochemical properties, lipophilicity, drug-likeness, and pharmacokinetics of chloroquine (CQ) and hydroxychloroquine (HCQ) based on their absorption, distribution, metabolism, and excretion (ADME) characteristics.

Entry	Chloroquine (CQ)	Hydroxychloroquine (HCQ)
Physicochemical Properties, Lipophilicity and Drug-Likeness		
Molecular weight (g/mol)	319.87	335.87
No. heavy atoms	22	23
No. arom. heavy atoms	10	10
Fraction Csp3	0.50	0.50
No. rotatable bonds	8	9
No. H-bond acceptors	2	3
No. H-bond donors	1	2
Molar Refractivity	97.41	98.57
TPSA (Å^2^)	28.16	48.39
Consensus Log *P*_o/w_	4.15	3.29
Lipinski’s Rule	Yes	Yes
Bioavailability Score	0.55	0.55
PAINS	0 alert	0 alert
**Pharmacokinetics**		
Gastrointestinal absorption	High	High
BBB permeant	Yes	Yes
P-gp substrate	No	No
CYP1A2 inhibitor	Yes	Yes
CYP2C19 inhibitor	No	No
CYP2C9 inhibitor	No	No
CYP2D6 inhibitor	Yes	Yes
CYP3A4 inhibitor	Yes	Yes
Log Kp (cm/s)	−4.96	−5.81
	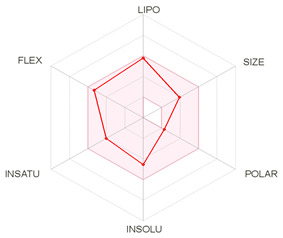	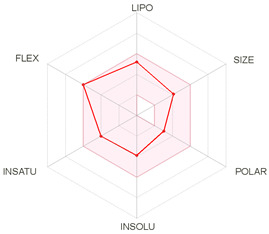

ADME, absorption, distribution, metabolism and excretion; LIPO (lipophilicity), −0.7 < XLOGP3 < +5.0; SIZE, 150 g/mol < MV < 500 g/mol; POLAR (polarity), 20 Å^2^ <TPSA < 130 Å^2^; INSOLU (insolubility), 0 < Log S (ESOL) < 6; INSATU (insaturation), 0.25 < fraction Csp3 < 1; FLEX (flexibility), 0 < number of rotatable bonds < 9; TPSA, topological polar surface area; CYP, cytochrome P450. The colored zone is the suitable physicochemical space for oral bioavailability.

## Data Availability

Data available in a publicly accessible repository that does not issue DOIs. Publicly available datasets were analyzed in this study.

## Sample Availability

Samples of the compounds are not available from the authors.
